# Surgical Essays

**Published:** 1818-03

**Authors:** 


*Surgical Essays.* By Astley Cooper, F.R.S. and Benjamin Travers, F.R.S. Part I. with numerous Plates. pp. 300. London, 1818.

The founders and supporters of public hospitals, those truly benevolent and *Christian* institutions, had little idea of the extent of the benefits which thence flow to *society at large.* Numerous as are the sufferers which *there* find an asylum during the pangs of disease, and experience the kindness, the humanity, and the skill of the medical officers, they form but a drop in the ocean, compared with those scattered over the earth, and who indirectly participate in the practical knowledge acquired in those inimitable schools of instruction and medical science, through the medium of the medical and surgical pupils there educated. But a great desideratum yet remained. The *pupils* of these institutions are incapacitated, from their ages and various other circumstances, for the important office of recording, analysing, and arranging the mass of materials which every day present themselves to their view. This requires the hand as well as the head of a *Master;* and truly happy are we to see the good work commencing and radiating from various centres, and thus diffusing the beams of science through every avenue and ramification of the profession. This indeed was a duty more incumbent on the superior medical officers of public hospitals to perform, than the daily routine of visits. They are the depositaries of practical knowledge, *ex officio;* but they only hold it in trust for the benefit of their less fortunate brethren, who anxiously look up to them for all great improvements in the science. It is true, they are not *compelled* to disseminate or communicate the knowledge they have thus acquired, and it is painful to reflect that few of them, *comparatively speaking,* have been generous enough to do so voluntarily; but, as the brave are always the most gentle, so the truly learned and experienced in the profession are always the most liberal; and the volume before us is an incontestible proof of this position. What can an Astley Cooper gain by the drudgery of publication? Does *his* reputation require any additional wings?—We may answer, that he can only gain the gratitude of the profession, and the blessings of the sick man, who knows not to whom he is indebted for the preservation of his life, but whose blessing shall be laid up—"where moth doth not corrupt, nor thieves break through and steal."

"Homines ad deos nullâ re propius accedunt, quam salutem hominibus dando." Cicero.

But to our subject. This is a work in every way worthy of a Cooper and a Travers. They have wisely determined to confine their reports to the *useful* in preference to the *wonderful*; to—

"—a narration that includes more of the common than the rare; for it is neither in the contemplation nor desire of the Editors to promulgate *marvellous* cases. The *singularity* of a case may be a good reason for its publication, but its *importance* is a better; and, in general, the greater its singularity, the less its importance." *Preface*, p. xii.

How often has this important truth flashed on our minds! How seldom has it proved the guide of either individuals or societies!—The work consists of six papers, three by each of the Editors. The first is on Dislocations, by Mr. Cooper; the second, on Iritis, by Mr. Travers; the third is the highly interesting Case of Ligature of the Aorta, by Mr. Cooper; the fourth, on Phymosis and Paraphymosis, by Mr. Travers; the fifth, on Exostosis, by Mr. Cooper; the last, on Wounds and Ligatures of Veins, by Mr. Travers. Of each of these, excepting one, we shall endeavour to give some account. The excepted paper is on *Iritis*, by Mr. Travers, which is so singularly interesting and important, that we mean to make it the subject of a *short* separate analysis on a future occasion. It is a fine specimen of accurate observation and legitimate analytical induction, tending to stamp an intrinsic value on every thing that flows from the same source.

1. *Dislocations.* Mr. Cooper, in his usual plain but energetic manner, forcibly points out the importance of *articular* anatomy, and the disgrace, not to say ruin, which may result from ignorance on this subject. He justly observes, that students will

"—often dissect the muscles of a limb with great neatness and minuteness, and then throw it away without any examination of the ligaments, the knowledge of which, in a surgical point of view, is of infinitely greater importance; and from hence arise the numerous errors of which they are guilty when they embark in the practice of their profession." P. 2.

To give any thing like a connected view of a paper in which there is scarcely a superfluous syllable, would be impossible in the limits of an analysis; and therefore we must confine ourselves to a few extracts. This is of the less consequence, as the work only wants *annunciation* to excite the general demand for its entire perusal. The following passage is long, but it is too important to be condensed, and will offer a fair example of the valuable information which the reader is to expect from the pen of Mr. Cooper.

" *Means of Reduction.*—The means employed for the reduction of dislocations are either constitutional or mechanical; it is generally wrong to employ force only, as it becomes necessary to use it in such a degree as to occasion violence and injury, and it will, in the sequel, be shewn, that the most powerful mechanical means fail, when unaided by constitutional. The power of the muscles, in the first instance, is to be duly appreciated, which forms the principal cause of resistance. The means to be employed for this purpose, are, to produce a tendency to syncope, and sometimes fainting itself, by the abstraction of a quantity of blood, and by placing the patient in a warm bath to occasion a similar feeling. If the blood be removed quickly, by a large orifice, it is known that fainting is more readily produced, and a hot bath from 100° to 110° will often not produce syncope, unless blood has been previously drawn.

" But of late years I have practised another mode of lowering the action of the muscles, by exhibiting nauseating doses of tartarized antimony. This, given in repeated doses, produces sickness, but not vomiting; emetics have been recommended, and there is no doubt but the state of nausea which they produced is useful, but the vomiting is, in itself, of no use, for as soon as the nauseating effect is produced, the muscles lose their tone, and dislocations can be reduced with comparatively less effort, and at a more distant time from the accident, than can be effected in any other way.—Two cases are related in the following pages: one from Mr. Norwood, Surgeon, Hertford, the other from Mr. Thomas, apothecary to St. Luke's Hospital; in which, by the combination of bleeding, warm bath, and nauseating doses of tartarized antimony, dislocations were reduced at a period from the accident, greater than I have ever known in any other example. One of these cases occurred at Guy's, and the other at St. Thomas's Hospital, at the time these gentlemen were officiating as dressers (See Cases of Dislocation on the Ilium).

" The effect of opium I have never tried, but it would probably be useful from its power of diminishing the nervous influence.

" The reduction of the bone is to be attempted after lessening the powers of the muscles, by making an extension of the limb, by fixing one bone and drawing the other towards its socket. One great cause of failure, in the attempt to reduce dislocations, arises from insufficient attention to not fixing that bone in which the socket is placed. As, for example, in attempting to reduce a dislocation of the shoulder, if the scapula be not fixed, or one person pulls at the scapula and two at the arm, the scapula is necessarily drawn with the os humeri, and the extension is very imperfectly made; the one bone, therefore, must be as firmly fixed as the other is extended.

"The force may be applied either by the exertion of assistants, or by a compound pulley, but the object is to extend the muscles by gradual, regular, and continued force; the pulley, in cases of difficulty, should always be resorted to; its force may be directed by the surgeon's mind; but when assistants are employed, their exertions are sudden, violent, and often ill directed, and the force is more likely to produce laceration of parts, than to restore the bone to its situation. Their efforts are also often uncombined, and their muscles necessarily fatigue, as those of the patient whose resistance they are employed to overcome.

"In dislocation of the hip-joint, pullies should always be employed, and in those dislocations of the shoulder which have remained long unreduced, they should always be resorted to. I do not mean to doubt of the possibility of reducing dislocation of the hip by the aid of men, but to point out the inferiority of this mode to pullies. Most writers on surgery have hinted at the use of pullies, but they have not duly appreciated them: my good master, Mr. Cline, whose professional judgment no man can deny, always strongly recommended them.

"During the attempt to reduce luxations, the surgeon should always endeavour to obtain a relaxation of the stronger muscles. The limb should therefore be kept in a position between flexion and extension, as far as it can be. Who has not seen, in the attempt to reduce a compound fracture, in the extended position of a limb, the bone incapable of being brought in apposition under the most violent efforts, quickly replaced by an intelligent surgeon, who immediately directed the limb to be bent, and the muscles to be placed in a comparative state of relaxation.

"A difference of opinion prevails, of whether it is best to apply the extension on the dislocated bone, or on the limb below. M. Boyer, who has long taken the lead in surgery in Paris, prefers the latter mode. As far as I have had an opportunity of observing, it is generally best to apply the extension to the bone which is dislocated. There are exceptions to this, however, in the dislocation of the shoulder, which I generally reduce by placing the heel in the axilla, and by drawing the arm at the wrist in a line with the side of the body, as when the arm is placed close to the side, the pectoral muscle and the latissimus dorsi are brought into a state of relaxation, and they form a powerful opposition when the arm is carried far from the side.

"Great advantage is derived in the reduction of dislocations, from attending to the patient's mind; the muscles opposing the efforts of the surgeon, by acting in obedience to the will, may have that action suspended, by directing the mind to other muscles. Several years ago, a surgeon, in Blackfriars Road, asked me to see a patient of his with a dislocated shoulder, which had resisted the various attempts he had made at reduction. I found the patient in bed, with his right arm dislocated; I sat down on the bed by his side, placed my heel in the axilla, and drew the arm at the wrist; the dislocated bone remained unmoved. I said, rise from your bed, Sir; he made an effort so to do, whilst I continued my extension, and the bone snapped into its socket: for the same reason, a slight effort, when the muscles are unprepared, will succeed in reduction of dislocation, after violent measures have failed." P. 25.

Mr. Cooper next lays down admirable, though concise instructions, for the diagnosis and reduction of the individual dislocations. In these instructions the accurate anatomist and expert surgeon are every where conspicuous, as well they may be. Cases, in elucidation, are clearly but succinctly narrated. We shall quote one as a specimen.

"I was desired to visit a man, aged 28 years, who, by the overturn of a coach, had dislocated his left hip more than five weeks before, and who had been declared not to have a dislocation, although the case was extremely well marked. His leg was full two inches shorter than the other; his knee and foot turned inwards, and the inner side of the foot rested opposite to the malleolus internus of the other leg. The thigh was slightly bent towards the abdomen, and the knee was advanced over the other thigh. The head of the thigh-bone could be distinctly felt upon the dorsum of the ilium; and when the two hips were compared, the natural roundness of the dislocated side had disappeared. I used only mechanical means in my attempts at reduction, and although I employed the pullies, and varied the direction of repeated extensions, I could not succeed in replacing the bone, and this person returned to the country with the dislocation unreduced." P. 35.

In the section on the fractures of the os innominatum, which are liable to be mistaken for dislocations, Mr. C. displays his usual accuracy of observation, and perspicacity of ideas. In half a dozen lines he conveys a clearer notion of things, than others would do in a whole page. But we must refer to the work itself; for, happily, this is a volume which cannot be analysed, on account of the intrinsic value and concentration of every part. The plates, though lightly executed, express with ease and accuracy the subject which they are designed to represent. Those, in particular, showing the modes of applying the apparatus for the reduction of hip-joint dislocations, are peculiarly satisfactory. The *subject* of dislocations is to be continued in the Second Part.

2. The second Paper in the Volume is on *Iritis,* by

Mr. Travers, which we shall reserve for a short separate Analysis in a future Number of this Journal. We therefore pass on to Mr. Cooper's

*Case of Ligature in the Aorta.* Mr. C. fears that the title of this paper may impress the reader with an idea that nothing could justify him in performing the operation. He is quite right. We have heard this observation several times made, and we have invariably repelled the charge. We are not among the cold and calculating surgeons, who would risk incurring a particle of popular clamour, to save the life of a fellow-creature, by attempting what was not sanctioned by written or oral authority. In this unsuccessful enterprize, Mr. Cooper has gained more real and permanent glory, than in all the operations he ever performed. But we are far from thinking the attempt unsuccessful, *in the end*, though it was so in the *beginning*. The derangement of parts, and the constitutional irritation that resulted thence, were such as precluded almost the hope of success. But the object is gained, the knowledge of the possibility of effecting the arrest of blood through the aorta, and that without any material injury to the parts through which the operation was carried, or shock to the vascular system, by the sudden arrest of so immense a current of blood. Every day impresses us more and more with the admiration of the wonderful—the almost omnipotent powers of Nature, in compensating for the losses or accidents to which our frames are subjected. Has not the experiment of Dr. Parry proved, that when a carotid artery was tied, new shoots sprouted out from the cardiac portion of the artery, traversed round the ligature, and dipped into the excommunicated portion, thus carrying on, through many small channels, the current that previously ran through one? What are we to expect after this? In respect to the abdominal aorta, when we survey the inosculations of the mammary and epigastric, the superior and inferior mesenteries, the lumbar arteries, &c. we cannot, for a moment, hesitate to believe that the mere obstruction of circulation in the descending aorta would be got over by the powers of Nature; and that it is principally the violence of the operation itself that we have to dread. This indeed is almost proved by a case of natural obstruction in the aorta brought forward by Mr. Cooper, and which was observed in the Hotel-Dieu, in 1789. The thoracic arteries were found so enlarged, that they could be felt running down the sides of the chest tortuous and dilated.

The aorta, immediately beyond its arch, was contracted to the size of a writing quill; and the case altogether clearly demonstrated, that the greater part of the blood usually conveyed by means of the aorta through the thorax, is capable of finding a circuitous course by the branches of the subclavian and intercostal arteries. It is also well known that Mr. Cooper has, several times, passed ligatures round the aortæ of dogs, and found the blood was readily transmitted by anastomosing vessels to the posterior extremities of the animal. We now come to this interesting and melancholy case.

C. H. 38 years of age, had fallen against the corner of a chest thirteen months previously, and received a violent blow in the left groin. On the following day the thigh was much swelled and discoloured; but after a confinement of three weeks, the limb returned to its natural size, and he resumed his employment, and worked, though with pain and difficulty, till within a fortnight of his entering the hospital, 9th of April 1817; when there appeared a swelling in the groin, partly above and partly below Poupart's ligament, with an obscure pulsation, and concluded to be aneurismal. The swelling was now diffused, with several large veins crossing its surface, and accompanied with much pain on pressure. In three days the swelling increased to double its former size, and the pulsation became less distinct. The tumour was so situated that it was impossible to tie the artery above the sac without cutting into the abdomen. Various temporary measures were put in force, but at length ulceration began, and on the 20th of May, hæmorrhage made its appearance. On the 25th, at nine o'clock at night, the case was desperate, and death was ready to snatch its victim. Anxious to avoid opening the abdomen, in order to secure the aorta near its bifurcation, Mr. C. determined, if possible, to pass a ligature around the artery *from within the aneurismal sac.* With this view, he made a small incision upon the tumour, about two inches above Poupart's ligament, and through a small opening in the sac, passed down his finger to feel for the aperture of the artery, but found only a chaos of broken coagula, and that the artery was destroyed within the parietes of the aneurism. This attempt therefore failed. As Mr. Cooper was quitting the patient's bedside, a sentiment of generous pity and manly resolution overcame all other considerations, and he nobly determined to tie the aorta, and thus afford the only ray of hope, and the only chance of saving his patient from *instant* dissolution.

Here is one of those traits of magnanimity which ennoble human nature, and which shed a conviction over the soul of man, that he is yet destined for "another and a better world." This "pleasing awful thought" is only alloyed by the bitter reflection, that there are men who, insensible to the feelings of humanity and the loud and eloquent appeal of Nature, could coolly condemn as rash, and almost impious, this truly Christian effort to turn aside the dart which had already sped from the hand of death towards the unfortunate victim! "Homo solus aut Deus aut Dæmon!"

*Operation.* "The patient's shoulders were slightly elevated by pillows, in order to relax, as much as possible, the abdominal muscles; for I expected that a protrusion of the intestines would produce embarrassment in the operation, and was greatly gratified to find that this was prevented by their empty state, in consequence of the involuntary evacuation of fæces; and here let me remark, that I should, in a similar operation, consider it absolutely necessary, previously to empty the bowels by active aperient medicines.

"I then made an incision, three inches long, into the linea alba, giving it a slight curve to avoid the umbilicus; one inch and a half was above, and the remainder below the navel; and the inclination of the incision was to the left of the umbilicus in this form [ ⟩ ]. Having divided the linea alba, I made a small aperture into the peritoneum, and introduced my finger into the abdomen; and then, with a probe-pointed bistoury, enlarged the opening into the peritoneum to nearly the same extent as that of the external wound. Neither the omentum nor the intestines protruded; and during the progress of the operation, only one small convolution projected beyond the wound.

"Having made a sufficient opening to admit my finger into the abdomen, I then passed it between the intestines to the spine, and felt the aorta greatly enlarged, and beating with excessive force. By means of my finger nail I scratched through the peritoneum on the left side of the aorta, and then gently moving my finger from side to side, gradually passed it between the aorta and spine, and again penetrated the peritoneum on the right side of the aorta. I had now my finger under the artery, and by its side I conveyed the blunt aneurismal needle, armed with a single ligature behind it; and my apprentice, Mr. Hey, drew the ligature from the eye of the needle to the external wound; after which the needle was immediately withdrawn.

"The next circumstance, which required considerable care, was the exclusion of the intestine from the ligature, the ends of which were brought together at the wound, and the finger was carried down between them, so as to remove every portion of the intestine from between the threads: the ligature was then tied, and the ends left hanging from the wound. The omentum was drawn behind the opening as far as the ligature would admit, so as to facilitate adhesion; and the edges of the wound were brought together by means of a quilled suture and adhesive plaster.

"During the time of the operation, the fæces passed off involuntarily, and the patient's pulse, both immediately, and for an hour after the operation, was 144 in the minute. He was ordered thirty drops of tincture of opium and camphorated mixture, and the involuntary discharge of fæces soon after ceased. I applied my hand to his right thigh immediately after the operation, and he said that I touched his foot; so that the sensibility of that leg was very imperfect.

"For the following particulars I am indebted to Mr. Cox, one of my apprentices.

"At midnight his pulse was 132.

26th. "At one o'clock in the morning, the patient complained of heat in the abdomen, but he felt no pain upon pressure; he said that his head felt hot, and that he had pain in the shoulders; his lower extremities, which were cold soon after the operation, were now regaining their heat; his body was in other parts covered with a cold sweat. The sensibility of the lower extremities has been very indistinct since the operation.

"At two o'clock, he felt so comfortable from his medicine that he wished to have more of it, and ten drops of tincture of opium were given him; his legs were wrapped in flannel, bottles of hot water were applied to the feet, and he then said that the heat of his belly was lessened.

"At six o'clock the sensibility of his limbs was still imperfect.

"At eight o'clock. A. M. he expressed himself as feeling quite comfortable; he however passed no urine, and had no evacuations; his right limb was warmer than the left, and the sensibility was returning.

"At noon the temperature of the right limb was 94, that of the left or aneurismal limb 87 $\frac{1}{2}$.

"At one o'clock, P. M. Mr. Cooper visited him; and as he walked up the ward, he appeared much gratified at seeing his patient, who was at the point of death the evening before, and who was now adjusting his bed-clothes, and smiled as Mr. C. approached the bed.

"At three o'clock, after a fit of coughing, the man was much alarmed with the idea of the thread having slipped into the wound: it was a false alarm; but, to prevent the idea of its recurrence, it was fastened to a quill: soon after this he complained of pain in the abdomen; it was not very severe, nor did it last long; readily yielding to fomentations. As he had no evacuation, he was ordered an enema.

"At six o'clock, P. M. he vomited soon after the glyster had been administered; the heat of the right leg was 96, that of the left or diseased limb 87 $\frac{1}{2}$

' At nine in the evening he took half a glass of port wine in warm water, which he immediately rejected; he complained of pain in the loins; his pulse was 104, and feeble; he was very restless; and had an involuntary discharge of fæces.

" Eleven at night, his pulse was 100 and weak; he still vomited.

27th. At 7, A. M. the report was that he had passed a restless night; the vomiting had returned at intervals; his pulse was 104, weak, and fluttering; he complained of pain all over his body, more particularly in his head; and the carotids beat with considerable force; he had great anxiety expressed in the countenance, was very restless, and the urine dribbled from him with some degree of pain at the end of the penis.

" At eight o'clock, A. M. the aneurismal limb appeared livid, and felt cold, more particularly around the aneurism; but the right leg remained warm.

" At eleven o'clock his pulse was 120, and weak; he appeared to be sinking. To the questions which were put to him he did not return any answer; he appeared to have an uneasiness about the heart, as he kept his hand upon the left breast. He died at eighteen minutes after one, P. M. having survived the operation forty hours.

" After being informed of his death, I requested Mr. Brookes, of Blenheim Street, to attend with me at the inspection of the body. Mr. Travers, surgeon of St. Thomas's Hospital, Mr. Stocker, apothecary of Guy's, and a large concourse of medical students attended the examination.

" When the abdomen was opened, we found not the least appearance of peritoneal inflammation, excepting at the edges of the wound. The omentum and intestines were free from any unnatural colour; the edges of the wound were glued together by adhesive inflammation, excepting at the part at which the ligature projected. We were much gratified to find that the ligature had not included any part of the omentum or intestine: the thread had been passed around the aorta, about three quarters of an inch above its bifurcation, and about an inch, or rather more, below the part where the duodenum crossed the artery. Upon carefully cutting open the aorta, a clot of more than an inch in extent was found to have sealed the vessel above the ligature; below the bifurcation, another, an inch in extent, occupied the right iliac artery, and the left was sealed by a third, which extended as far as the aneurism; all were gratified to observe the artery so completely shut in forty hours. The aneurismal sac, which was of a most enormous size, reached from the common iliac artery to below Poupart's ligament, and extended to the outer side of the thigh. The artery was deficient from the upper to the lower part of the sac, which was occupied by an immense quantity of coagulum.

" The neck of the thigh bone had been broken within the capsular ligament, and had not been united." p. 124.

It is evident here that the patient did not die from visceral inflammation, nor the shock of the operation, but solely from the want of circulation in the aneurismal limb, owing to the too advanced stage of the disease.

Our limits prevent us at present from noticing the other papers in this invaluable volume, which few will fail to peruse.

